# Supplementary material for: Stacking Model‐Based Classifiers for Dealing With Multiple Sets of Noisy Labels
Source: Biom J. 2025 Mar 12;67(2):e70042. doi: 10.1002/bimj.70042 (PMC11898607; doi:10.1002/bimj.70042)
Supplement: Supplementary file 1 — Supporting Information [file BIMJ-67-e70042-s001.zip › density-based-ensemble-model-main/competitors_real_data/netlab-master/netlab-master/help/demsom1.htm]

Netlab Reference Manual demsom1


# demsom1

## Purpose

Demonstrate SOM for visualisation.

## Synopsis

```
demsom1
```

## Description

This script demonstrates the use of a
SOM with a two-dimensional grid to map onto data in
two-dimensional space. Both on-line and batch training algorithms
are shown.

## See Also

`som`, `sompak`, `somtrain`

---

**Pages:**
Index


---

Copyright (c) Ian T Nabney (1996-9)
